# Impact of new rural cooperative medical scheme on the equity of health services in rural China

**DOI:** 10.1186/s12913-018-3288-2

**Published:** 2018-06-22

**Authors:** Jianhua Chen, Hengjin Dong, Hai Yu, Yaming Gu, Tao Zhang

**Affiliations:** 1 Sir Run Run Shaw Hospital, Zhejiang University School of Medicine, Hangzhou, China; 2 Center for Health Policy Studies, School of Public Health, Zhejiang University School of Medicine, Hangzhou, China; 3 Zhejiang University School of Medicine, Hangzhou, China; 4 Department of Primary Health Care Delivery System, Health and Family Planning Commission of Zhejiang Province, Hangzhou, China; 5 Ningbo Municipal Center for Disease Control and Prevention, Ningbo, Zhejiang Province China

**Keywords:** Rural residents, Equity, Health services utilization, New cooperative medical scheme

## Abstract

**Background:**

To explore the equity in health services in the rural areas, by studying the rural residents in two counties of Ningbo City, Zhejiang.

**Methods:**

Multi-stage stratified cluster random sampling method was performed to draw the study sample. Trained investigators conducted face to face interviews using a questionnaire. Rural residents were stratified into 5 income groups. Centralized index (CI) and risk ratios (RR) were used to assess the equity in health services and the impact of the New Cooperative Medical Scheme (NCMS).

**Results:**

The centralized index of the 2 weeks prevalence, two-week visiting rate and chronic disease prevalence among different income groups was − 0.0264, − 0.0076 and − 0.0160, respectively, while that of the hospitalization rate was 0.0006. The highest NCMS coverage rate, adjusted risk of disease and catastrophic health expenditure (CHE) rates were observed in lowest income groups (92.86, 4.94 and 32.21%, respectively. Two-week prevalence, chronic diseases prevalence and RR showed a declining trend with increasing income levels.

**Conclusion:**

NCMS has improved the service availability and reduced the economic burden of diseases. However, its impact on reducing the economic risk of illness and promoting equity in health services has not been significant.

**Electronic supplementary material:**

The online version of this article (10.1186/s12913-018-3288-2) contains supplementary material, which is available to authorized users.

## Background

The World Health Organization has identified universal health coverage as a key approach to reduce equity gaps, and social health insurance is the recommended strategy to achieve universal health coverage. Social health insurance generally refers to health insurance schemes provided by governments for its citizens, especially for the low and middle-income populations. Universal coverage may take different forms based on the specific sociocultural and economic context of a country. The mechanisms may vary from single-payer government systems to mixed models comprising a variety of financing mechanisms. Currently, the basic medical insurance systems in China comprise of Urban Employee Basic Medical Insurance, Urban Residents Basic Medical Insurance, The New Cooperative Medical Scheme (NCMS) and Medical assistance system, which basically covers the entire population. The equity of utilization of medical services among various coverage groups has always been the focus of attention. For example, Zhang Xing applied concentration index to explore the equity of medical service utilization under different social medical insurance schemes. They found that better outpatient planning may improve the equity of out-patient medical treatment, and that the level of hospitalization equity were related to medical insurance reimbursement, the design of medical insurance system and the level of economic development [1]. In view of the lack of equity, other scholars have suggested expansion of the coverage of social medical insurance and implementation of a compensation mechanism based on prepayment [2].

Specifically, the NCMS is a Chinese government-led health insurance scheme that aims to make medical insurance more affordable for the rural peasantry. The scheme was launched in the year 2003 and has improved the access to medical insurance among its target population [3, 4]. The core values and mission of NCMS is to promote equity in health care, whereby every member of the society has equal access to health care services irrespective of the socioeconomic status [5, 6].

Improvement in population health not only depends on the quantum of investment in health resources, but also on the efficiency of health resource allocation, as well as on the management and utilization of health services by the communities. The World Health report (2013) proposed three dimensions of national health coverage: breadth (population coverage), depth (quantity and quality of health service) and height (economic risk protection), which consist of the performance of the equity of health services [7]. Much of the research has focused on the breadth of coverage of the NCMS; the other two dimensions, i.e., the depth and height dimensions health services have not been adequately researched [8–10].

The health care system in the Zhejiang province has seen rapid development since 1990; an urban health system and a three-tier system for rural preventive health care has been established. The NCMS is well implemented in the province and currently provides medical insurance cover to all rural residents. Nonetheless, there are still some deficiencies in the NCMS and the rural health care system at large. Ran et al. found that NCMS alone is not enough to address the health needs of the rural residents and that it may cause further impoverishment, especially of the lower income groups [11]. A study by SG Shen suggested that the financing among individual rural residents in NCMS was unfair [12]. Furthermore, Han et al. reported that the rural residents’ health services utilization equity is generally good since the implementation of NCMS; however, they identified the need to pay more attention to poor rural residents [13]. NCMS has helped improve access to health services; however, its effect on reducing the economic burden of disease on the low-income rural population has been rather limited [14]. The method of concentration index has been used to analyze the effect of NCMS in China. The results showed that rural residents’ equity of inpatient utilization is improved by NCMS; however, the gap between high-income residents and low-income residents with respect to utilization of outpatient services has widened [15]. Based on the concentration index analysis, the new rural cooperative medical system has helped improve the fairness of medical resource utilization among low-income populations according to another study conducted in2015 [16]. In the present study, we used a multi-stage stratified cluster random sampling method to investigate the distribution and equity of health services among rural residents in Zhejiang province. A centralized index was used to assess health needs of populations at different economic levels. Equity in health care utilization was examined to generate scientific evidence and the rationale for greater funding of health insurance.

## Methods

### Sample selection

Zhejiang is one of the more economically developed provinces in China, With an area of 101,800 km^2^ and a population of 54,890,000 as of 2013. The 11 divisions of Zhejiang are subdivided into 90 county-level divisions (36 districts, 20 county-level cities, 33 counties, and one autonomous county). Yuyao and Fenghua Counties of Ningbo City, which have relatively good economic and more rural population were chosen as the study sites. Multi-stage stratified cluster random sampling method was employed to collect representative sample. In each county, two townships were randomly selected according to the economic income; two villages were selected in each township. In order to ensure the consistency of the subjects, we used the sampling method employed in the national health service survey. A total of 400 households were surveyed; the number of households sampled in each village depended on the size of the villages. Members of the sampled households were required to be resident in Ningbo City, Zhejiang Province for at least the last 6 months. The sampling methodology is consistent with that used in the Chinese fourth national health services survey, and is the standard used for the World Bank-funded projects, for example, for evaluation of basic health services in rural areas and the new cooperative medical scheme evaluation.

### Definitions

In this survey, “Illness within two-weeks” was defined from the perspective of the perceived health service needs of the respondents and was self-reported. Specifically: 1, self-perceived illness in the last 2 weeks immediately preceding the data of interview for which diagnostic and therapeutic services were accessed at a health unit; 2, self-perceived illness in the last 2 weeks immediately preceding the data of interview for which the respondent resorted to self-medication; 3, Self-perceived illness in the last 2 weeks immediately preceding the data of interview for which the respondent did not receive diagnostic and therapeutic service at a health unit and also did not self-medicate or received complementary therapy, but for which the respondent remained off work or in bed for at least 1 day.

Occurrence of any of the three scenarios was considered as “Illness within two-week”.

Income: Income was defined as per capita net income of farmers, which was calculated as: total income - household expenses - taxes and fees - productive fixed assets depreciation - gift for people residing within the rural area.

Two-week prevalence: The illness status of respondents in the last 2 weeks was used to estimate the prevalence of two-week.

Two-week visiting rate: Utilization of institutional health care services at any level of health service delivery, for a self-perceived episode of illness in the first 2 weeks was used to estimate the two-week visiting rates.

Hospitalization rate: Hospitalization among the respondents within the previous year was used to estimate hospitalization rates.

Catastrophic health expenditure (CHE): CHE is defined as a percentage of out-of-pocket expenses which are payments made by a household for their health services without compensations from a third party [17]. 40% is defined as the pre-defined percentage for CHE in the present study.

### Survey instrument

The *Questionnaire for health services for rural areas* was administered for data collection on the following variables: (1) Need for health services among rural residents: demographic characteristics, self-evaluation of health, prevalence of injury, disability and health risk factors; (2) Demand for and utilization of health services among rural residents: treatment services, satisfaction demand for health services and the reason for dissatisfaction, utilization of public health services, maternal and child health care, emergency and inpatient services, personal medical expenses; (3) rural health insurance system: composition and coverage of health insurance, scope and level of compensation, operation of health insurance system (4) Degree of satisfaction of residents: including the attitude towards the health system, service delivery, medical insurance coverage and level.

### Study groups

Subjects were grouped according to income level, which is the most commonly used method to study the status of health services in the world. Potential differences and unfairness of health status and health services utilization among different income groups were compared. Additionally, the household contribution towards health care financing in the different income groups was assessed. The data was sorted according to net per capita income of residents and categorized into five income groups depending on the number of rural residents: the lowest income group, 2nd low-income group, middle income group, the 4th income group and the highest income group.

### Field data collection and quality control

Face to face interviews were conducted in the households with support from the local health bureau. The leadership team was established, and the activities were coordinated out of the respective Disease Prevention and Control Centers at the counties (CDC). Health bureau took the lead in the planning, organizing, training, implementation and quality control of the survey. Informed oral consent was obtained from respondents; all members of the household were interviewed one by one according to the scope of the items included in the questionnaire. The investigation was held by the medical staff of the township health center. All investigators had participated in the previous two surveys. Training of investigators was centralized to ensure the uniformity in, and the quality of field activities. The instructors of investigations were responsible for the investigation, supervision, inspection and quality control [18].

### Data processing and analysis

Double data entry was performed in parallel in EpiData 3.1 (Epidata Association; Odense, Denmark). After performing data consistency checks, data output was obtained in Excel format. Stata 12.0 (Stata Corporation; College Station, Texas) was used for statistical analyses. Statistical methods including ANOVA analysis, chi square test and concentration index (CI). Statistical significance level was set as 0.05.

Concentration index represents a common approach for assessment of equity in health services research. Equity of health services for rural residents with different income level in this study. Generally, CI is stratified by socioeconomic status, which is sensitive to the demographic changes among different socio-economic groups, and further reflects the impact of different socio-economic conditions on health inequity [12]. CI curve represents the distribution of percentage (a specific percentage of population health variables (h-i)/total cumulative of population health variables), from low to high ranking of income (γ-i).

Concentration index is 2 times of area enclosed by concentrated curve and diagonal curve. In the present study, least squares method was used to produce a linear fit. CI values ranged between − 1 and 1. For health, “CI = -1” indicates health resources focus on the people who lived in lowest strata of the society, while “CI = + 1” represents that health resources focus on the groups with the highest strata.

For disease, a negative CI indicates that the disease is concentrated in the lower social strata, while positive CI indicates that the disease is concentrated in the higher social strata. A CI approaching 0 is indicative of an equitable distribution of health and disease.

## Results

### Basic information

A total of 4900 (2473 men [50.47%] and 2472 women [49.47%] rural residents stratified into 5 income levels were interviewed (Table 1). The number of subjects in the lowest, 2nd, middle, 4th and the highest income groups was 981 (20.02%), 1205 (24.59%), 805 (16.43%), 1135 (23.16%) and 774 (15.80%), respectively.

**Table 1:** Basic characteristics of study subjects

	Total	Income groups					F/χ^2^	*P*
		Lowest	2nd	Middle	4th	Highest		
No. of peasants, N (%)	4900 (100.00)	981 (20.02)	1205 (24.59)	805 (16.43)	1135 (23.16)	774 (15.80)		
Male, N (%)	2473 (50.47)	477 (48.62)	600 (49.79)	411 (51.06)	583 (51.37)	402 (51.94)	χ^2^ = 2.70	0.609
Average age (y)	46.60	56.36	47.69	43.36	42.37	42.12	F = 88.17	< 0.001*
Annual per capita income (RMB)	16,908.43	4615.10	9213.39	12,584.93	17,338.74	48,335.19	F = 490.16	< 0.001*
Consumption per capita (RMB)	9515.35	5203.00	6826.14	7782.01	11,018.41	18,766.35	F = 390.47	< 0.001*
Medicine expenditure per capita (RMB)	962.32	1297.37	832.13	750.16	918.41	1025.39	F = 5.95	< 0.001*
NCMS coverage rate (%)	91.53	92.86	92.53	92.42	90.93	88.24	χ^2^ = 15.96	0.003*
Villager’s pay for NCMS (RMB)	74.67	64.39	75.35	74.81	74.74	87.03	F = 25.39	< 0.001*
NCMS reimbursement rate (%)	80.61	81.96	80.00	86.34	77.53	78.42	χ^2^ = 105.01	< 0.001*

*: *P* < 0.05, rate was calculated based on the number of subjects

*NCMS* New Cooperative Medical Scheme

No significant intergroup differences were observed with respect to the gender mix in different income groups (*P* = 0.609). The average age was 46.6 years; there were significant differences in the average age in different income groups (*P* <  0.001); the average age in the lowest income group was 14.2 years which was higher than that in the highest income group. The annual per capita income was 16,908.4 RMB, and significant difference (*P* <  0.001) was observed in the annual per capita income between different income groups; annual per capita income in the highest income group was 10.4 times higher than that in the lowest income group. Annual per capita cost of health care was 962.3 RMB; there were significant inter-group differences in this respect, too (*P* <  0.001), the lowest income group of health care expenses was 1297.37 RMB; The NCMS coverage was 91.5%, with significant inter-group differences in coverage between the different income groups (*P* = 0.003); the lowest income group had the highest coverage rate of NCMS, (92.8%), while the highest income group had the lowest coverage (88.2%). The annual villager’s pay for NCMS was 74.6 RMB; there were significant differences with respect to the NCMS annual cost (*P* <  0.001) between the different income groups. NCMS reimbursement rate was 80.6% with significant inter-group differences (*P* <  0.001); the highest rate was observed in the middle-income group.

### Health service needs and utilization

The two-week prevalence declined with increasing revenue, and the prevalence in different income groups was significantly different (χ^2^ = 109.22, *P* <  0.001), the prevalence in the lowest and highest income groups was 31.70 and 17.83%, respectively; CI was − 0.0264 ± 0.0068, which shows that the health service needs tended to be higher in the low-income groups (Table 2). Two-week visiting rate was significantly different between the different income groups (χ^2^ = 21.34, *P* <  0.001), and the rates for the lowest and highest income group were 6.52 and 4.26%; the corresponding CI was − 0.0076 ± 0.0036. Non hospitalization rates for the lowest and the highest income groups were 0.61 and 0.26%, with the ratio being 2.35:1 in favor of the lowest income groups. Prevalence of chronic diseases in the lowest and highest income groups was 28.03 and 14.99%, respectively; the CI is − 0.0160 ± 0.0065.

**Table 2:** Concentration index of health care utilization in Zhejiang province

	Total	Income groups					χ^2^	*P*	CI	95% CI	*t*	*P*
		Lowest	2nd	Middle	4th	Highest						
Two-week prevalence (%)	20.61	31.70	21.58	16.15	15.07	17.83	109.22	< 0.001	−0.0264	[−0.0398, − 0.0131]	−3.88	< 0.001
Two-week visiting rate (%)	4.20	6.52	3.57	4.47	2.64	4.26	21.34	< 0.001	−0.0076	[− 0.0146, − 0.0006]	−2.12	0.034
Non-clinical visit rate (%)	1.29	2.34	1.41	1.49	0.53	0.65	16.70	0.002	−0.0079	[− 0.0109, − 0.0048]	−5.06	< 0.001
Chronic disease prevalence (%)	19.57	28.03	21.83	15.16	16.12	14.99	77.39	< 0.001	−0.0160	[−0.0288, − 0.0031]	−2.44	0.015
Hospitalization rate (%)	3.43	4.99	3.65	4.10	2.38	1.94	17.52	0.002	0.0006	[−0.0053, 0.0066]	0.21	0.837
Non-hospitalization rate (%)	0.53	0.61	0.66	0.62	0.44	0.26	1.95	0.752	−0.0001	[−0.0020, 0.0020]	−0.05	0.959

*: *P* < 0.05, rate was calculated based on the number of subjects

*CI* Concentration index, *95% CI* 95% confidence interval

### Economic disease burden

Risk ratio (RR) can be used to measure the economic risk of disease in different income groups. It refers to the ratio of per capita health care costs between specific populations and the sample population, while the other factors remain unchanged.

Table 3 shows that RR reduced with the increase in the annual per capita net income of farmers. Although the RR has increased in the highest income group, it is still less than the value for the lowest income group, which indicates that low-income populations use more medical services due to their greater needs.

**Table 3:** Economic risks of the disease in different income groups

Income group	Drug expenditure per capita (RMB)	GDP per capita (RMB)	RR	Adjusted RR
Lowest	1297.37	4615.10	1.35	4.94
2nd	832.13	9213.39	0.86	1.59
Middle	750.16	12,584.93	0.78	1.05
4th	918.41	17,338.74	0.95	0.93
Highest	1025.39	48,335.19	1.07	0.37

*RR* risk ratio, *GDP* gross domestic product

After using “per capita net income of sample population/per capita net income of specific populations” as a correction factor to eliminate the influence of income on RR, a greater degree of decline in adjusted RR values was observed. The RR in the low-income group (4.94) was 13.3 times that in the highest income group (0.37), which indicates that although low-income population uses more health services, they remain exposed to a greater financial risk of illness at the same time.

Table 4 indicates that the payment ability for medicine expenditure reduced with the increase in income level (F = 67.05, *P* <  0.001), by using household as the research unit. Among these, the proportion of the lowest and the highest income groups was 21.33 and 4.51%, respectively; range ratio was 4.73:1, which implies that the low-income group households face relatively greater economic risks of disease.

**Table 4:** Income and expenditure by income group

Income group	Income	Consumption expenditure	Medicine expenditure	Food expenditure	Non-food expenditure	Payment ability rate for medicine expenditure	Catastrophic health
	(RMB annually)	(RMB annually)	(RMB annually)	(RMB annually)	(RMB annually)	(%)	expenditure rate (%)
Lowest	13,551.43	14,225.21	3033.63	6055.71	8169.50	21.33	32.21
2nd	30,687.27	21,956.88	2377.05	10,349.07	11,607.81	10.83	13.44
Middle	47,363.08	28,470.13	2681.02	13,210.54	15,259.60	9.42	11.80
4th	59,632.25	39,157.93	2766.77	13,981.88	25,176.05	7.07	6.87
Highest	174,595.60	64,111.06	2891.60	20,688.28	43,422.79	4.51	5.81
Total	59,432.45	32,121.97	2729.99	12,420.15	19,701.82	8.50	14.20

The results showed that the percentage of CHE showed a decreasing trend with increases of income (chi-square test results χ2 = 360.21, *P* <  0.001). Among them, the percentage in the lowest and highest income groups were 32.21 and 5.81%, respectively; range ratio was 5.54:1.

## Discussion

Health service utilization is impacted by various variables as the needs of health care among the residents are not same. Even in the presence of wide differences in the extent of health care utilization between different population groups, an accurate assessment of inequity and its quantification is often challenging. For example, the age-distribution and health care needs are not consistent; therefore, the “vertical equity” analysis can be considerably more difficult. According to the principle of “horizontal equity”, population health needs should be consistent with the utilization of health services, which allows for fair and clear judgment about health services, and quantification of inequity by using CI.

In this study, we found that the utilization of inpatient services gradually decreased with increase in the economic status. Furthermore, both the utilization of in-patient services and the non-hospitalization rates in the lowest income group were at least two times more than that in the richest group; this suggests that the lowest income population subgroup tends to have a greater need for health services than the higher-income groups. This is attributable to low reimbursement rates for outpatient services and high hospital reimbursement rates. The CI of hospitalization rate indicates a good equity (0.0006), which is lower than that for Shanxi (0.31), Gansu (0.31) and Zhejiang (0.37) in 2003 [19]. The utilization of in-patient services has greatly improved over the last decade.

With respect to the outpatient services, the two-week visiting rate of the lowest income groups was up to 6.52% and the corresponding CI was − 0.0076, which implies that the equity in utilization of outpatient health services was relatively worse than that for the in-patient services. The full implementation of NCMS has improved the utilization of health services, but has also caused a concomitant increase in the health expenditure. Furthermore, some participants did not utilize health services due to the economic burden, which was not taken into account seriously for the present. The resilience of the low-income groups against economic risks of disease is less than that of the high-income groups, which not only relates to the greater need for health care in the former, but also to the lower amount of compensation that they are entitled to. Although NCMS reduces the incidence of CAH, the impact of NCMS, especially on the low-income groups has been limited (the percentage of lowest income group was 32.21%). The low-income group continues to experience the “high need, low utilization, high-burden, low-income” phenomenon. Our study suggests that the equity of health services has not significantly improved for rural residents.

Given the instability of income of the rural residents, we cautiously propose to improve and diversify the level of funding for NCMS. For example, the county can be divided into several levels according to the income and people pay a certain proportion of income and set the corresponding reasonable reimbursement, to improve equity among rural residents.

In recent years, Zhejiang Province has introduced interventions for the medical market, such as social security card, which led to more convenient real-time settlement of medical expenses. In addition, centralized procurement of basic drugs and establishment of unified payment settlement system has brought down the price of drugs and ensured the safety of medication. By institutionalizing the classification of diagnosis and treatment (mainly using the differentiated medicare payment, medical price leverage and standardize the transfer procedures of diagnosis), the framework for the “ailment in the community, serious illness into the hospital, rehabilitation back to the community” medical model has been established.

The present study has some limitations. Firstly, since the two counties (Yuyao and Fenghua County) belong to regions with relatively higher economic level, the results of this study may not be applicable to all regions. However, in this study we did not focus on the functional status of health services. Secondly, the study involves only two counties and employs a smaller sample size. Large scale studies with better sampling methodology (e.g., the WHO approved 30*7 clusters) may provide a more robust analysis of the health services. Thirdly, as the study was completed 5 years back, more recent data should also be included in the analysis. Fourthly, the equity of health services are liable to be impacted by rural basic economic system, disparities in regional development, different literacy levels for rural residents and other extraneous influences. Therefore, the determinants of equity in health services should be further investigated. In addition, under MSA/Catastrophic, patients are likely to demand more inpatient services which may further increase the number of impoverished households. Similarly, under the RMHC, patients would demand more primary health care and a few people may demand more inpatient services, which could, again, increase health expenditure and medical impoverishment.

Even with these limitations, some meaningful inferences may be drawn from our study. The utilization of rural health service appears to qualify the ‘fairness’ criteria, but inequities exist in utilization of outpatient health service among rural residents, and low-income rural residents need more health care. Equity in health service utilization plays an important role in minimizing the financial risk of illness and improvement of the new rural cooperative medical system.

## Conclusion

NCMS plays an important role in improving service availability and in reducing the economic burden of diseases; however, this effect is limited to reduction in economic risks while equity in health services has not significantly improved. NCMS policy should also be more equity-oriented to achieve its policy goal.

## Additional file

Additional file 1: Family Health Survey. (DOC 103 kb)

## Abbreviations

CI: Concentration index
MSA: Medical saving account
NCMS: New cooperative medical scheme
