# Supplementary material for: Impact of new rural cooperative medical scheme on the equity of health services in rural China
Source: BMC Health Serv Res. 2018 Jun 22;18:486. doi: 10.1186/s12913-018-3288-2 (PMC6013905; doi:10.1186/s12913-018-3288-2)
Supplement: Supplementary file 1 — Family Health Survey. (DOC 103 kb) [file 12913_2018_3288_MOESM1_ESM.doc]

**Table 1 General information questionnaire**

| **number** | **question** | **answer** |
| --- | --- | --- |
| 1 | How many people are there in your family? |  |
| 2 | How many persons lived in your family in the past six months? |  |
| 3 | How many persons did not live at home in the past six months? |  |
| 4 | How far is the nearest medical unit to your home?   1. Less than 1 kilometre (2) one- (3) two- (4) three- (5) four- (6) more than 5 kilometres |  |
| 5 | How long does it take from your home to the nearest medical unit (minutes)? |  |
| 6 | Annual household income? |  |
| 7 | How much did your family spend on living expenses in the previous year? |  |

**Table 2 Family health questionnaire**

| **Number code** | | **01** | **02** | **03** | **04** | **05** | **06** |
| --- | --- | --- | --- | --- | --- | --- | --- |
| **A.** Personal information | |  |  |  |  |  |  |
| 1 | **Name** |  |  |  |  |  |  |
| 2 | Relationship with the head of household  (1) head of household (2) spouse (3)Children (4)Grandchildren  (5) parents (6) grandparents (7) sisters and brothers (8) 0thers |  |  |  |  |  |  |
| 3 | Sex： (1)male (2)female |  |  |  |  |  |  |
| 4 | Nation: |  |  |  |  |  |  |
| 5 | Date of birth: (year) **(for example :1998）** |  |  |  |  |  |  |
| 6 | (month) |  |  |  |  |  |  |
| 7 | Marital status： (1)unmarried (2)In marriage (3)divorce (4)widowed (5)other |  |  |  |  |  |  |
| 8 | How much do you pay for NCMS each year? |  |  |  |  |  |  |
| **B. Disease information** | |  |  |  |  |  |  |
| 9 | Did you feel any discomfort in the last two weeks? (1)yes (2)no |  |  |  |  |  |  |
| 10 | Were you suffering from a chronic disease diagnosed by a doctor in the last six months?  (1) yes (2) no |  |  |  |  |  |  |
| 11 | Disease name |  |  |  |  |  |  |
| 12 | Disease coding |  |  |  |  |  |  |
| 13 | Time of diagnosis: (1) Half an year ago (2) Within six months |  |  |  |  |  |  |
| 14 | Has treatment been taken within six months? (1) yes (2)no |  |  |  |  |  |  |
| 15 | Did treatment take place six months ago? (1) yes (2) no |  |  |  |  |  |  |
| 16 | Have you been hospitalized for illness in the past 12 months ? (1)yes (2)no |  |  |  |  |  |  |
| 17 | How many times did you stay in the hospital? |  |  |  |  |  |  |
| 18 | In the past 12 months, was there a time when the doctor recommended hospitalization but you did not stay in the hospital? (1) yes (2) no |  |  |  |  |  |  |
| 19 | If so, how many times did this happen? |  |  |  |  |  |  |
| 20 | If more than two times, was it for the same reason? (1) yes (2) no |  |  |  |  |  |  |
| 21 | The reason for refusing hospitalization: (1) not necessary (2) no effective treatment (3) financial difficulties (4) poor hospital services (5) lack of time (6) lack of hospital bed (7) others |  |  |  |  |  |  |

**Table 3 Medical questionnaire**

| member code | | **01** | **02** | **03** | **04** | **05** | **06** |
| --- | --- | --- | --- | --- | --- | --- | --- |
| 1 | What was the main discomfort in the last 2 weeks?  (1) fever (2) pain (3) diarrhea (4) cough (5) palpitation  (6) others (7) no symptom |  |  |  |  |  |  |
| 2 | Extent of the sickness: (1) not serious (2) common (3) serious |  |  |  |  |  |  |
| 3 | What was your disease? (disease name) |  |  |  |  |  |  |
| 4 | (disease code) |  |  |  |  |  |  |
| 5 | Did you receive any treatment? (1) yes (2) no |  |  |  |  |  |  |
| 6 | If no, what was the main reason for not receiving treatment ?  (1) self-perceived mild nature of illness (2) financial constraint  (3) lack of time (4) traffic inconvenience (5) no effective measures  (6) others |  |  |  |  |  |  |
|  | |  |  |  |  |  |  |
| 7 | How did you treat it? (1) self-care  (2) consulted a doctor two weeks ago, and continuing with treatment  (3) consulted a doctor in the last two weeks |  |  |  |  |  |  |
| 8 | How much is the total cost of medicine to treat the disease? |  |  |  |  |  |  |
| 9 | Who pays for these medical expenses?  (1) Personal/home account (2) Partial reimbursement or relief  (3) Full reimbursement or relief (4) out of pocket expenses for the entire treatment |  |  |  |  |  |  |

**Table4 Inpatient questionnaire**

| **Member code** | | **01** | **02** | **03** | **04** | **05** | **06** |
| --- | --- | --- | --- | --- | --- | --- | --- |
| 1 | Why are you in hospital? (1) disease (2) injury (3) Rehabilitation  (4) Physical examination (5) Childbirth (6) other |  |  |  |  |  |  |
| 2 | Disease name |  |  |  |  |  |  |
| 3 | Disease code |  |  |  |  |  |  |
| 4 | The hospital admission time: (year) |  |  |  |  |  |  |
| 5 | (month) |  |  |  |  |  |  |
| 6 | What kind of medical institution do you stay in?  (1) health service center (2) County people's hospital (3) County hospital of traditional Chinese medicine (4) Provincial hospital |  |  |  |  |  |  |
| 7 | How many days in the hospital? |  |  |  |  |  |  |
| 8 | How much was the total cost of this hospitalization? |  |  |  |  |  |  |
| 9 | How much was the total reimbursement? |  |  |  |  |  |  |
| 10 | How much were the expenses for travel, meals and escort fees for this hospitalization? |  |  |  |  |  |  |
